# Supplementary material for: Pyruvate Dehydrogenase Kinase 1 inhibition mediated oxidative phosphorylation enhancement in cartilage promotes osteoarthritis progression
Source: BMC Musculoskelet Disord. 2023 Jul 20;24:597. doi: 10.1186/s12891-023-06585-6 (PMC10357736; doi:10.1186/s12891-023-06585-6)
Supplement: Supplementary file 1 — Additional file 1. [file 12891_2023_6585_MOESM1_ESM.zip › BP.Gsea.1653623667859/GOBP_GLYCOSYL_COMPOUND_CATABOLIC_PROCESS.html]

Details for gene set GOBP\_GLYCOSYL\_COMPOUND\_CATABOLIC\_PROCESS[GSEA]

|  || Dataset | 55457\_collapsed\_to\_symbols.cls.cls #OA\_versus\_normal.cls.cls #OA\_versus\_normal\_repos |
| Phenotype | cls.cls#OA\_versus\_normal\_repos |
| Upregulated in class | OA |
| GeneSet | GOBP\_GLYCOSYL\_COMPOUND\_CATABOLIC\_PROCESS |
| Enrichment Score (ES) | 0.42045408 |
| Normalized Enrichment Score (NES) | 1.4811989 |
| Nominal p-value | 0.036319613 |
| FDR q-value | 0.37901437 |
| FWER p-Value | 1.0 |
Table: GSEA Results Summary

  

Fig 1: Enrichment plot: GOBP\_GLYCOSYL\_COMPOUND\_CATABOLIC\_PROCESS      
 Profile of the Running ES Score & Positions of GeneSet Members on the Rank Ordered List

  

| SYMBOL | TITLE | RANK IN GENE LIST | RANK METRIC SCORE | RUNNING ES | CORE ENRICHMENT || 1 | ABHD10 | "abhydrolase domain containing 10, depalmitoylase [Source:HGNC Symbol;Acc:HGNC:25656]" | 437 | 0.690 | 0.0372 | Yes |
| 2 | ENPP4 | ectonucleotide pyrophosphatase/phosphodiesterase 4 [Source:HGNC Symbol;Acc:HGNC:3359] | 822 | 0.563 | 0.0654 | Yes |
| 3 | GUSB | glucuronidase beta [Source:HGNC Symbol;Acc:HGNC:4696] | 904 | 0.541 | 0.1149 | Yes |
| 4 | DERA | deoxyribose-phosphate aldolase [Source:HGNC Symbol;Acc:HGNC:24269] | 975 | 0.523 | 0.1634 | Yes |
| 5 | APOBEC3C | apolipoprotein B mRNA editing enzyme catalytic subunit 3C [Source:HGNC Symbol;Acc:HGNC:17353] | 1355 | 0.450 | 0.1803 | Yes |
| 6 | NUDT1 | nudix hydrolase 1 [Source:HGNC Symbol;Acc:HGNC:8048] | 1425 | 0.439 | 0.2203 | Yes |
| 7 | FUCA1 | alpha-L-fucosidase 1 [Source:HGNC Symbol;Acc:HGNC:4006] | 1506 | 0.428 | 0.2582 | Yes |
| 8 | NAGA | alpha-N-acetylgalactosaminidase [Source:HGNC Symbol;Acc:HGNC:7631] | 1674 | 0.402 | 0.2867 | Yes |
| 9 | GBA3 | glucosylceramidase beta 3 (gene/pseudogene) [Source:HGNC Symbol;Acc:HGNC:19069] | 1767 | 0.388 | 0.3196 | Yes |
| 10 | AICDA | activation induced cytidine deaminase [Source:HGNC Symbol;Acc:HGNC:13203] | 1825 | 0.381 | 0.3544 | Yes |
| 11 | APOBEC3G | apolipoprotein B mRNA editing enzyme catalytic subunit 3G [Source:HGNC Symbol;Acc:HGNC:17357] | 1917 | 0.370 | 0.3855 | Yes |
| 12 | ADA2 | adenosine deaminase 2 [Source:HGNC Symbol;Acc:HGNC:1839] | 1955 | 0.367 | 0.4205 | Yes |
| 13 | APOBEC1 | apolipoprotein B mRNA editing enzyme catalytic subunit 1 [Source:HGNC Symbol;Acc:HGNC:604] | 2695 | 0.280 | 0.3919 | No |
| 14 | MTAP | methylthioadenosine phosphorylase [Source:HGNC Symbol;Acc:HGNC:7413] | 3324 | 0.219 | 0.3657 | No |
| 15 | GBA | glucosylceramidase beta [Source:HGNC Symbol;Acc:HGNC:4177] | 3327 | 0.219 | 0.3880 | No |
| 16 | APOBEC3F | apolipoprotein B mRNA editing enzyme catalytic subunit 3F [Source:HGNC Symbol;Acc:HGNC:17356] | 3603 | 0.193 | 0.3866 | No |
| 17 | DPYD | dihydropyrimidine dehydrogenase [Source:HGNC Symbol;Acc:HGNC:3012] | 3830 | 0.173 | 0.3869 | No |
| 18 | GLA | galactosidase alpha [Source:HGNC Symbol;Acc:HGNC:4296] | 4172 | 0.140 | 0.3748 | No |
| 19 | APOBEC2 | apolipoprotein B mRNA editing enzyme catalytic subunit 2 [Source:HGNC Symbol;Acc:HGNC:605] | 4279 | 0.130 | 0.3800 | No |
| 20 | APOBEC3B | apolipoprotein B mRNA editing enzyme catalytic subunit 3B [Source:HGNC Symbol;Acc:HGNC:17352] | 5089 | 0.065 | 0.3238 | No |
| 21 | ADA | adenosine deaminase [Source:HGNC Symbol;Acc:HGNC:186] | 8105 | -0.163 | 0.1065 | No |
| 22 | CDA | cytidine deaminase [Source:HGNC Symbol;Acc:HGNC:1712] | 8324 | -0.180 | 0.1081 | No |
| 23 | XDH | xanthine dehydrogenase [Source:HGNC Symbol;Acc:HGNC:12805] | 8366 | -0.183 | 0.1238 | No |
| 24 | UPB1 | beta-ureidopropionase 1 [Source:HGNC Symbol;Acc:HGNC:16297] | 9609 | -0.284 | 0.0565 | No |
| 25 | PNP | purine nucleoside phosphorylase [Source:HGNC Symbol;Acc:HGNC:7892] | 9657 | -0.288 | 0.0826 | No |
| 26 | APOBEC3A | apolipoprotein B mRNA editing enzyme catalytic subunit 3A [Source:HGNC Symbol;Acc:HGNC:17343] | 10550 | -0.378 | 0.0522 | No |
| 27 | UPP1 | uridine phosphorylase 1 [Source:HGNC Symbol;Acc:HGNC:12576] | 12013 | -0.595 | 0.0000 | No |
| 28 | CDADC1 | cytidine and dCMP deaminase domain containing 1 [Source:HGNC Symbol;Acc:HGNC:20299] | 12267 | -0.667 | 0.0492 | No |
Table: GSEA details [plain text format]

  

Fig 2: GOBP\_GLYCOSYL\_COMPOUND\_CATABOLIC\_PROCESS      
 Blue-Pink O' Gram in the Space of the Analyzed GeneSet

  

Fig 3: GOBP\_GLYCOSYL\_COMPOUND\_CATABOLIC\_PROCESS: Random ES distribution      
 Gene set null distribution of ES for **GOBP\_GLYCOSYL\_COMPOUND\_CATABOLIC\_PROCESS**
